# *ASXL1* mutations predict inferior molecular response to nilotinib treatment in chronic myeloid leukemia

**DOI:** 10.1038/s41375-022-01648-4

**Published:** 2022-07-28

**Authors:** Lioba Schönfeld, Jenny Rinke, Anna Hinze, Saskia N. Nagel, Vivien Schäfer, Thomas Schenk, Christian Fabisch, Tim H. Brümmendorf, Andreas Burchert, Philipp le Coutre, Stefan W. Krause, Susanne Saussele, Fatemeh Safizadeh, Markus Pfirrmann, Andreas Hochhaus, Thomas Ernst

**Affiliations:** 1 Abteilung Hämatologie und Internistische Onkologie, Klinik für Innere Medizin II, Universitätsklinikum Jena, Jena, Germany; 2 Department of Hematology, Oncology, Hemostaseology and Stem Cell Transplantation, Medical Faculty, RWTH Aachen University, Aachen, Germany; 3 Klinik für Innere Medizin, Schwerpunkt Hämatologie, Onkologie und Immunologie, Universitätsklinikum Marburg, Marburg, Germany; 4 Medizinische Klinik, Hämatologie und Onkologie, Charité - Universitätsmedizin Berlin, Berlin, Germany; 5 Medizinische Klinik 5 (Hämatologie und Internistische Onkologie), Universitätsklinikum Erlangen, Erlangen, Germany; 6 III. Medizinische Klinik, Medizinische Fakultät Mannheim der Universität Heidelberg, Mannheim, Germany; 7 Institut für Medizinische Informationsverarbeitung, Biometrie und Epidemiologie – IBE, Ludwig-Maximilians-Universität, München, Germany

**Keywords:** Chronic myeloid leukaemia, Cancer genomics

## Abstract

Gene mutations independent of *BCR::ABL1* have been identified in newly diagnosed patients with chronic myeloid leukemia (CML) in chronic phase, whereby mutations in epigenetic modifier genes were most common. These findings prompted the systematic analysis of prevalence, dynamics, and prognostic significance of such mutations, in a clinically well-characterized patient population of 222 CML patients from the TIGER study (CML-V) by targeted next-generation sequencing covering 54 myeloid leukemia-associated genes. In total, 53/222 CML patients (24%) carried 60 mutations at diagnosis with *ASXL1* being most commonly affected (*n* = 20). To study mutation dynamics, longitudinal deep sequencing analysis of serial samples was performed in 100 patients after 12, 24, and 36 months of therapy. Typical patterns of clonal evolution included eradication, persistence, and emergence of mutated clones. Patients carrying an *ASXL1* mutation at diagnosis showed a less favorable molecular response to nilotinib treatment, as a major molecular response (MMR) was achieved less frequently at month 12, 18, and 24 compared to all other patients. Patients with *ASXL1* mutations were also younger and more frequently found in the high risk category, suggesting a central role of clonal evolution associated with *ASXL1* mutations in CML pathogenesis.

## Introduction

Chronic myeloid leukemia (CML) is characterized by the *BCR::ABL1* gene fusion, which codes for a constitutively active tyrosine kinase [1]. Inhibition of this aberrant kinase via imatinib – a selective tyrosine kinase inhibitor (TKI), revolutionized the treatment of CML [2]. Second-generation TKIs such as nilotinib improved treatment further, resulting in earlier and higher response rates while offering lower risk of disease progression [3]. Whilst some patients show resistance to TKI treatment and are at high risk of progression, for a large number of patients living without treatment has become a realistic aim if durable deep molecular remission under TKI treatment has been achieved [4, 5]. According to previous studies, myeloid-leukemia associated mutations occur in addition to *BCR::ABL1* in approximately 30% of newly diagnosed CML patients [6–8]. Mutations were most commonly identified in epigenetic modifier genes such as *ASXL1*, *DNMT3A*, and *TET2*. Two retrospective studies suggested an inferior response to TKI of CML patients harboring mutations of epigenetic regulator genes [7, 9]. Large population studies revealed an increased prevalence of such variants also in elderly people lacking hematological disorders [10–12], suggesting a rate of 10% in people older than 65 years but only 1% in people younger than 50 years of age [11]. However, our recent findings of *ASXL1* mutations in children and young adults with CML indicate that the existence of such mutations can be disease- rather than age-related in CML patients [13].

To advance our understanding of the role of mutations in addition to *BCR::ABL1*, the study presented here aimed to evaluate their prevalence and dynamics in a large, well-characterized patient population in an ongoing prospective clinical trial. Furthermore, the clinical impact of such mutations was investigated to understand their effect on molecular response during TKI treatment.

## Patients and methods

### Patients

The patient cohort consisted of 222 randomly selected chronic phase CML patients treated within the prospective German TIGER study (CML-V; NCT01657604). All patients received nilotinib based TKI therapy. Patients’ characteristics are provided in Table 1 and Supplementary Table 1. Median age was 52 years (range, 18–79 years), 140 (63%) were male. 116 patients (52.3%) received nilotinib monotherapy and 106 patients (47.7%) a combination with nilotinib and pegylated interferon alpha 2b (pegIFN) according to the study protocol. All procedures were in accordance with the standards of the ethics committee and the Declaration of Helsinki. Informed consent for participation in the trial and molecular analysis was obtained from all patients.

**Table 1:** Patients’ characteristics.

	Total	No mutations	All mutations		
				*ASXL1* mutations	Other mutations
Age at diagnosis (years)					
Median (range)	52 (18–79)	52 (18–79)	54 (19–78)	47 (24–68)	56 (19–78)
Sex (*n*, %)					
Male	140 (63%)	108 (64%)	32 (60%)	15 (75%)	17 (52%)
Female	82 (37%)	61 (36%)	21 (40%)	5 (25%)	16 (48%)
EUTOS score (*n*, %)					
Low risk	203 (91%)	157 (93%)	46 (87%)	13 (65%)	33 (100%)
High risk	19 (9%)	12 (7%)	7 (13%)	7 (35%)	0 (0%)
*BCR::ABL1* transcript type (n, %)					
b2a2	86 (39%)	69 (41%)	17 (32%)	5 (25%)	12 (36%)
b3a2	100 (45%)	72 (43%)	28 (53%)	11 (55%)	17 (52%)
b2a2 + b3a2	36 (16%)	28 (17%)	8 (15%)	4 (20%)	4 (12%)
Total	222	169	53	20	33

*EUTOS* European Treatment and Outcome Study.

### Sample preparation

Peripheral blood samples of all patients were collected at diagnosis and every three months on treatment. Genomic DNA (gDNA) was isolated from peripheral blood leukocytes after red cell lysis using the QIAamp DNA Mini Kit (Qiagen, Hilden, Germany) according to the manufacturer’s recommendations. Additionally, buccal swab samples were used where available for the isolation of constitutional DNA to verify the somatic origin of mutations. The RNeasy Mini Kit (Qiagen) or TRIzol reagent (Invitrogen, Carlsbad, CA, USA) was used according to the manufacturer’s recommendations to extract total leukocyte RNA after red cell lysis from at least 20 ml of peripheral blood. As previously published, complementary DNA synthesis was performed using random hexamer primers and Moloney murine leukemia virus reverse transcriptase (Invitrogen) [14] or SuperScript IV VILO Master Mix (Thermo Fisher Scientific, Waltham, MA, USA) according to manufacturer’s instructions.

### Real-time quantitative PCR and Sanger sequencing

For this study, patients’ molecular response to therapy was measured after 12, 18, 24, and 36 months of therapy, unless the patient had died or dropped out. In total 657 samples were analyzed. LightCycler technology (Roche Diagnostics, Mannheim, Germany) or the Quantstudio5 (Thermo Fisher) system was employed for *BCR::ABL1* transcript quantification as described previously [15, 16]. Molecular response was assessed by RT-qPCR determining the ratio of *BCR::ABL1* to *ABL1* transcripts and reported on the International Scale (IS) [17]. Treatment response was reported as log level reduction of *BCR::ABL1*^IS^ according to the European LeukemiaNet (ELN) recommendations: major molecular response (MMR) ≤ 0.1%, MR^4^ ≤ 0.01%, MR^4.5^ ≤ 0.0032%, MR^5^ ≤ 0.001% [18, 19]. Patients with failure criteria according to ELN 2013 recommendations [18] were analyzed by Sanger sequencing for the presence of *BCR::ABL1* kinase domain mutations.

### Next-generation sequencing

Next-generation sequencing (NGS) of 54 genes frequently mutated in various entities of myeloid malignancies was performed using the TruSight Myeloid Panel on the Mini Seq platform (Illumina, San Diego, CA, USA) with 8 samples per run. In total, 522 samples were analyzed; 222 samples at diagnosis with follow-up analyses for 100 patients after 12, 24, and 36 months of therapy. Mean amplicon coverage ranged from 1762–21,465 reads/amplicon. The collected data was analyzed using the VariantStudio Software (version 2.0; Illumina, San Diego, CA, USA) with the hg19 genome build and a sensitivity level of 5% variant allele frequency (VAF). Variants detected at low VAF (<5%) were only included if the same variant was found with a frequency above 5% in at least one sample of the respective patient. As recommended by the developers, the software’s own PASS filter was used. Synonymous coding mutations and intronic polymorphisms were excluded. Variants with an allele frequency of ≥1% in the general population were also excluded. In accordance to recent recommendations of Branford et al. [8] the Combined Annotation Dependent Depletion (CADD; version1.3) [20] was used with a threshold of ≥20 to filter missense variants; additionally, only those variants predicted to be damaging by at least 3 of 4 prediction tools were included. We used the open access tools Polymorphism Phenotyping v2 (PolyPhen-2) [21] and dbNSFP version 4.0a [22] for SIFT [23], MutationTaster [24] and FATHMM [25]. Missense variants considered to be germline by buccal-swap analysis (*n* = 5) or due to a VAF of 50% or consistent VAF of 50 or 100% over time (*n* = 6) which otherwise met the published criteria of clinically relevant mutations [8] were regarded separately. For the predictive analysis, the *ASXL1* c.1934dupG mutation was excluded based on frequent sequencing artifacts in this homopolymer area.

### Statistics

For statistical analysis, all timepoints were calculated from the day of randomization or from the day of the first visit when trial participation was confirmed. To be allocated to a certain time, a molecular analysis was performed within the ±3-months interval around that time. Any event worse than “no MMR”, that is disease progression or death from any cause within an interval, was rated as “no MMR” for this and any following interval. The probability of MMR was given as the proportion of patients with at least MMR or “no MMR” at 12, 18, and 24 months, according to their mutation status. The association between two categorical variables was assessed with Fisher’s exact test. Group comparisons with regard to continuous variables were performed with the Mann-Whitney U-Test. To investigate the influence of mutations on molecular status at 12, 18, and 24 months under consideration of other baseline variables, multiple logistic regression was used. All statistical tests were explorative. The level of significance was 0.05. Point estimations are given together with their 95% confidence interval (95%-CI). SAS version 9.4 was used for analysis.

## Results

### Molecular response

*BCR::ABL1*^IS^ was assessed at months 12, 18, and 24 for all 222 patients of this sub-cohort of the TIGER study. After one year of TKI treatment, 181 patients (82% of 222, 95%-CI: 76–86%) had achieved at least MMR, whilst 49% of patients showed a molecular response of MR^4^ or better (95%-CI: 43–56%), 29% had at least MR^4.5^ (95%-CI: 23–35%), and 5% were in MR^5^ (95%-CI: 3–9%). At 18 months, one patient had been lost to follow-up and three patients (rated as “no MMR”) had died. Thus, of 221 patients, 192 (87%, 95%-CI: 82–91%) had achieved at least MMR, whilst 54% (95%-CI: 48–61%) were assessed as MR^4^ or better and 32% (95%-CI: 26–39%) had achieved at least MR^4.5^. MR^5^ was achieved for 8% (95%-CI: 5–13%) of 221 patients. Before month 24, one more patient was lost to follow-up. At 24 months, of 220 patients, 193 (88%, 95%-CI: 83–91%) had achieved at least MMR. Proportions of patients with at least MR^4^, at least MR^4.5^, or MR^5^ were 59% (95%-CI: 52–65%), 38% (95%-CI: 32–45%), and 14% (95%-CI: 10–19%), respectively.

### Mutations at diagnosis

**Fig. 1:**
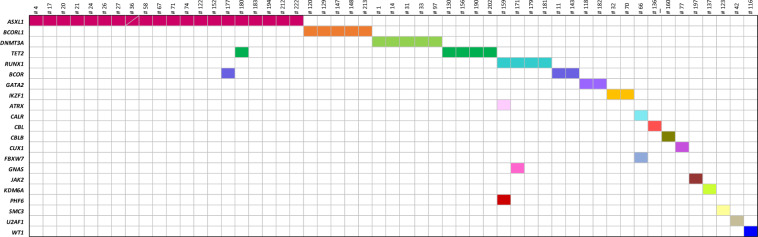
Landscape of somatic mutations in 222 chronic phase CML patients at diagnosis by targeted next-generation sequencing. Mutations were found in 53/222 patients (24%) with *ASXL1* being the most commonly affected gene (*n* = 20). Patients and genes are displayed in columns and rows, respectively. A unique color is assigned to each mutated gene. Bisected cell represents two variants in the same gene and patient.

At the time of diagnosis, 53 of 222 patients (24%) carried 60 mutations in addition to *BCR::ABL1*, affecting the genes *ASXL1*, *ATRX*, *BCOR*, *BCORL1*, *CALR*, *CBL*, *CBLB*, *CUX1*, *DNMT3A*, *FBXW7*, *GATA2*, *GNAS*, *IKZF1*, *JAK2*, *KDM6A*, *PHF6*, *RUNX1*, *SMC3*, *TET2*, *U2AF1*, and *WT1* (Fig. 1). In detail, 18 distinct nonsense mutations, 13 frameshift variants, 22 missense mutations, two inframe deletions and one inframe insertion were detected. *ASXL1* mutations were most frequently identified and were observed in 20 patients (9%). Six patients showed more than one additional mutation. This affected patient #36 (*ASXL1*, *ASXL1*), #66 (*CALR*, *FBXW7*), #159 (*RUNX1*, *ATRX*, *PHF6*), #171 (*RUNX1*, *GNAS*), #177 (*ASXL1*, *BCOR*) and #180 (*ASXL1*, *TET2*). Median age of patients carrying additional mutations to *BCR::ABL1* at diagnosis was 54 years (range, 19–78 years). Buccal swab samples were available for 13 patients and showed no or lower-level mutation frequencies, hence indicating a somatic origin of the respective mutations.

Apart from the mutations described above, 10 distinct missense variants were detected in 14 patients that were considered to be germline but otherwise met the published criteria of clinically relevant mutations [8] (Supplementary Table 1). The affected genes were *ASXL1*, *ATRX*, *CBL*, *IDH2*, *KDM6A*, *RUNX1*, *TET2*, *TP53*, and *ZRSR2*. These variants were found in all analyzed samples of the respective patients with a VAF of approximately 50% (*n* = 8) or 100% (*n* = 2) and/or were confirmed to be germline via buccal swab analysis if available (*n* = 5). Four patients not analyzed in follow-up carried a *TET2* variant with a VAF of approximately 50% that was otherwise considered clinically relevant.

### Mutation dynamics

**Fig. 2:**
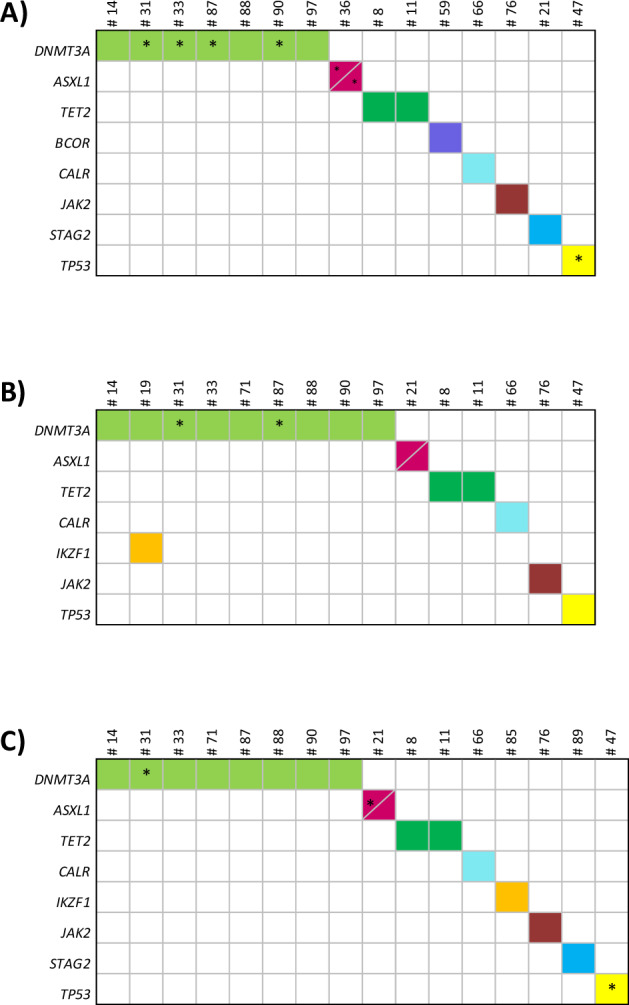
Prevalence of somatic mutations in 100 chronic phase CML patients during nilotinib treatment. Longitudinal deep sequencing analysis of serial samples after (**A**) 12, (**B**) 24, and (**C**) 36 months of therapy. *DNMT3A* mutations were most frequently identified. Patients and genes are displayed in columns and rows, respectively. A unique color was assigned to each mutated gene. A bisected cell represents two variants in the same gene and patient. Asterisks stand for low allele variants (<5% variant allele frequency).

Investigating follow-up samples of 100 randomly chosen patients of the cohort described above, we observed three mutational patterns: (I) eradication, (II) persistence, (III) emergence of mutations (Table 2a–c). Regarding mutation prevalence at month 12, 24, and 36 please refer to Fig. 2A–C.

**Table 2:** Dynamics of somatic mutations in 100 chronic phase CML patients at diagnosis and after 12, 24, and 36 months of nilotinib therapy.

(a) Variants detected only at diagnosis or suppressed and subsequently eradicated in follow-up. *ASXL1* L775X re-occurred at month 24 in patient #21. Samples with molecular response of at least MMR are shaded in green, no MMR is shaded in red. Low level allele variants (<5% variant allele frequency) are displayed in brackets. *n* = 19 patients.

(b) Variants detected in every analyzed sample of the respective patient. Samples with molecular response of at least MMR are shaded in green. Low level allele variants (<5% variant allele frequency) are displayed in brackets. *n* = 5 patients.

(c) Variants detected only in follow-up samples. Samples with molecular response of at least MMR are shaded in green, no MMR is shaded in red. Low level allele variants (<5% variant allele frequency) are displayed in brackets. *n* = 13 patients.

Most mutations were eradicated together with the *BCR::ABL1* clone under nilotinib therapy (pattern I, Table 2a). Nineteen patients showed this type of mutation pattern, most commonly affecting *ASXL1* with *n* = 12 detections. For most patients, the mutation was detected at diagnosis only and was undetectable during treatment. However, in patient #36 two *ASXL1* mutations detected at diagnosis were also found at month 12. After a year of nilotinib treatment the mutations had been suppressed to low VAFs of 2.7% and 2.3%, respectively (Table 2a). At this point the patient showed a *BCR::ABL1*^IS^ transcript level of 2.8%. Analysis of subsequent follow-up samples showed that *BCR::ABL1* as well as both *ASXL1* mutations were cleared which indicates that the *ASXL1* mutations were located in Ph+ cells. At month 24, patient #21 showed re-emergence of an *ASXL1* mutation initially detected at diagnosis (the month 12 sample was tested negative) with a *BCR::ABL1*^IS^ transcript level of 13% at that time (Table 2a). Another follow-up sample was analyzed at month 36. The *ASXL1* variant increased in frequency compared to month 24 and a *BCR::ABL1*^IS^ transcript level of 35% was assessed at this point. The patient underwent allogeneic stem cell transplantation. Though not included in NGS follow-up analysis, patient #194, who presented with an *ASXL1* mutation at diagnosis, showed an E255K *BCR::ABL1* mutation at months 12 and 18 as detected by Sanger sequencing (Supplementary Table 1).

In five patients mutations persisted (pattern II; Table 2b). Follow-up samples also revealed a reduction of the allele burden of four of these mutations (*DNMT3A* in all cases) compared to the level at diagnosis whereas the *CALR* mutation displayed a slightly increased VAF in follow up analyses. Since VAF was significantly above *BCR::ABL1* transcript levels in all patients, the *BCR::ABL1* translocation has most likely occurred on top of the preexisting mutation. Median age of these patients was 59 years (range, 42–71 years) at diagnosis.

Fifteen variants in 13/100 (13%) patients newly emerged in follow-up that had not been detected in the corresponding diagnostic samples (pattern III; Table 2c). Affected genes were *ASXL1*, *BCOR*, *DNMT3A*, *IKZF1*, *JAK2*, *STAG2*, *TET2*, and *TP53* with the highest prevalence in *DNMT3A* (*n* = 5). Since VAFs of these variants were significantly above *BCR::ABL1*^IS^ transcript levels in all patients, these mutated clones are considered to be *BCR::ABL1* negative. Patients gaining a mutation in follow-up had a median age of 56 years (range, 32–73 years) at diagnosis.

Data further suggests different mutational dynamics in follow-up samples in patients with more than one mutation. In patients #19 and #36 both mutations followed the same mutation patterns of eradication and emergence, respectively (Table 2a, c). Non-parallel mutation dynamics were observed in patients #11 (*BCOR* eradication; *TET2* emergence), #21 (*ASXL1* eradication/re-emergence, *ASXL1* and *STAG2* emergence), #66 (*FBXW7* eradication, *CALR* persistence) and #71 (*ASXL1* eradication, *DNMT3A* emergence) (Table 2a–c).

### Clinical impact of mutations

**Fig. 3:**
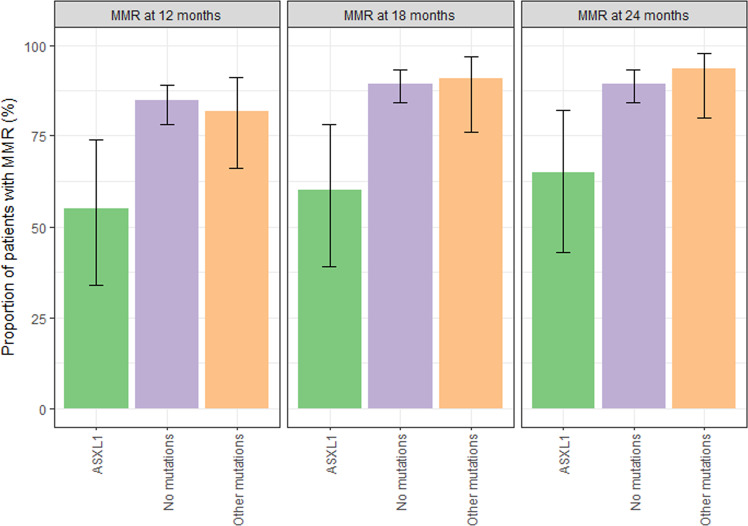
Proportion of patients with major molecular response (MMR) based on mutation status at diagnosis. Patients carrying an *ASXL1* mutation at diagnosis achieved significantly lower MMR probabilities than patients without any mutation, showing 55% (95%-CI: 34–74%) at 12 months (*p* = 0.0036), 60% (95%-CI: 39–78%) at 18 months (*p* = 0.0018), and 65% (95%-CI: 43–82%) at 24 months (*p* = 0.0076). Black vertical lines describe the 95% confidence intervals around the proportions indicated by the upper end of the bars. MMR Major molecular response.

In patients without mutation at diagnosis, the probabilities of achieving MMR or better were 85% (95%-CI: 78–89%) at 12 months, 89% (95%-CI: 84–93%) at 18 months, and 89% (95%-CI: 84–93%) at 24 months. With 82% (95%-CI: 66–91%) at 12 months, 91% (95%-CI: 76–97) at 18 months, and 94% (95%-CI: 80–98%) at 24 months the probabilities of MMR in the patients with mutations (excluding *ASXL1*) were not significantly different. In contrast, patients carrying an *ASXL1* mutation at diagnosis achieved significantly lower MMR probabilities than patients without any mutation (Fig. 3), showing 55% (95%-CI: 34–74%) at 12 months (*p* = 0.0036), 60% (95%-CI: 39–78%) at 18 months (*p* = 0.0018), and 65% (95%-CI: 43–82%) at 24 months (*p* = 0.0076). Investigating the categorizations “at least MR^4^“, “at least MR^4.5^”, and “at least MR^5^“, no statistically significant and clinically relevant difference between “no mutations” and either “ASXL1” or “other mutations” was found.

Using multiple logistic regression, apart from mutation status, no additional variable reported in Table 1 had a significant influence on the achievement of the molecular status “MMR or better” at 12, 18, or 24 months. The odds ratios of *ASXL1* as compared with “no mutations” were 0.222 (95%-CI: 0.084–0.589), 0.179 (95%-CI: 0.065–0.496), and 0.223 (95%-CI: 0.079–0.631) with increasing MMR proportions and time.

Table 1 displays patient characteristics distinguished by “no mutations” and “all mutations”, the latter separated in “*ASXL1* mutations” and “other mutations”. Any mutations at diagnosis occurred in 46/203 (22.7%) low risk patients according to the EUTOS score and in 7/19 (36.8%) high risk patients (n.s.). However, all mutations other than *ASXL1* were only identified in low-risk patients and accordingly, all seven mutations identified in 19 high-risk patients were *ASXL1* mutations. *ASXL1* mutations at diagnosis occurred in 13/203 (6.4%) low risk patients and 7/19 (38.8%) high risk patients (*p* = 0.0004). Patients with *ASXL1* mutation were more frequently at high risk than patients with other (*p* = 0.0005) or no mutations (*p* = 0.0012). Moreover, patients with *ASXL1* mutation were younger than patients with other mutations (*p* = 0.0380). No further associations between mutations and other patient characteristics were observed.

## Discussion

This study provides a prospective analysis of leukemia-associated gene mutations in newly diagnosed CML patients treated within a controlled clinical trial. Several retrospective studies found that mutations in leukemia-associated genes in addition to *BCR::ABL1* are not only a phenomenon seen in advanced or blast crisis CML but also in chronic phase CML [6–9]. As CML is primarily diagnosed in chronic phase, a better understanding of clonal molecular evolution may aid therapeutic decision-making, risk-stratification and management in the future.

At the time of diagnosis, patients most frequently carried *ASXL1* mutations (*n* = 20). *ASXL1* mutations are also commonly detected in other myeloid malignancies and are generally associated with a poor clinical outcome. As an epigenetic and transcriptional regulator, ASXL1 is involved in chromatin modifications and interacts with polycomb complex proteins as well as transcriptional activators and suppressors [26]. Recently, Yang et al. showed a gain-of-function in mice carrying *ASXL1* exon 12 mutations yielding gene truncation. The truncation resulted in more open chromatin and a dysregulated expression of genes critical for the self-renewal and differentiation of hematopoietic stem cells [27]. Moreover, as a component of the BAP1 histone H2A deubiquitinase complex, ASXL1 truncation increases its stability, strengthening the association of the BAP1 complex with chromatin, driving oncogenic gene expression [28].

*ASXL1* mutations have previously been observed in CML in adults [6–8] and also in children [13] where *ASXL1* was the only gene found to be mutated which may indicate a relevant role of such mutations in some cases. The study presented here provides novel evidence for an adverse response to TKI treatment of CML patients carrying mutant *ASXL1* as the presence of an *ASXL1* mutation at diagnosis was associated with a worse response to nilotinib treatment as measured by MMR achievement at month 12, 18, and 24. Mutations other than ASXL1 mutations at diagnosis did not affect molecular response during follow-up. Moreover, patients with *ASXL1* mutations were younger than patients with other mutations and more frequently at high risk than patients with other or no mutations. All mutations other than *ASXL1* were only identified in low risk patients. These findings indicate that ASXL1 mutations may have an active role in CML pathogenesis and clonal evolution.

Our data confirm previous findings which show that *ASXL1* mutations are rarely detected during MMR suggesting eradication of the mutation in most patients by TKI treatment [7]. However, we found a slower treatment response in CML patients carrying an *ASXL1* mutation at diagnosis despite mutation clearance in 11/12 of the affected patients in which follow-up samples were analyzed. This suggests that clonal evolution with *ASXL1* mutations on top of *BCR::ABL1* influences TKI susceptibility of leukemic cells and thus response to therapy. The molecular mechanism behind this phenomenon needs to be explored further. Recently, Wang et al. [28] identified a small molecule inhibitor which targets aberrant BAP1 catalytic activity in *ASXL1*-mutated leukemia, reversing oncogenic gene expression. In the future, combined or sequential treatment strategies could help CML patients with *ASXL1* mutations achieve better molecular responses.

Whilst the presence of multiple mutations per patient are commonly detected in blast crisis CML, acute myeloid leukemia (AML) and myelodysplastic neoplasms (MDS) [29–31], 47/53 (89%) of all affected patients in this study showed only one mutation in addition to *BCR::ABL1* at diagnosis indicating that the clonal architecture in CML might be less complex compared to other myeloid malignancies (Fig. 1). However, five patients of the cohort investigated in this study (#36, #66, #171, #177 and #180) showed two mutations and one patient (#159) showed three distinct mutations in addition to *BCR::ABL1* at diagnosis. Patient #159 also carried an Y253H *BCR::ABL1* mutation (month 12) as detected by Sanger sequencing and was the only patient in this cohort to develop blast crisis. The patient died 21 months after treatment initiation. As CML samples are increasingly sequenced to investigate causes of resistance, persistence, and relapse, future analyses may confirm that the number of additional mutations has clinical consequences, as seen in AML and chronic myelomonocytic leukemia (CMML), where the number of mutations detected is associated with a worse prognosis [32, 33]. The current study is based on targeted NGS, including a panel of 54 leukemia-associated genes, whereas whole exome or genome analyses might reveal additional mutations. As clinical datasets combined with sequencing information expand (e.g., within the HARMONY Plus Alliance; www.harmony-alliance.eu), a clearer picture of the impact of additional mutations may emerge.

Sequencing analyses of diagnosis samples and corresponding annual follow-up samples in 100 selected patients revealed various mutation patterns under therapy, including eradication (pattern I), persistence (pattern II), and emergence (pattern III). Regarding the eradication group, in 19/100 (19%) patients (at least one) additional mutation was detected at diagnosis that was subsequently undetectable under nilotinib treatment (Table 2a). As these mutations were not detectable in subsequent *BCR::ABL1* negative follow-up samples, it may be concluded that only *BCR::ABL1* positive cells harbored these additional mutations. One patient showed re-occurrence of a variant at month 24 with a concurrently increased *BCR::ABL1*^IS^ transcript level of 1.1%, supporting the notion that these additional mutations are located in *BCR::ABL1* cells. To further strengthen these findings, future analyses could employ error-corrected NGS to increase sensitivity further and confirm mutation concomitance.

Fifteen variants in 13/100 (13%) patients were only detected in follow-up samples. One of these mutations was the *JAK2* V617F mutation in patient #76 which has been previously described as uncommon in CML [34]. In some of these rare cases the diagnosis of a *JAK2* V617F-positive disease preceded the acquisition of the Ph-chromosome [35, 36]; in others the second myeloproliferative disease emerged in the remission phase of CML [37–39]. In the case presented here, the *JAK2* V617F mutation was not detected at diagnosis, indicating that it was either initially masked by the predominantly existing Ph+ cells or arose during nilotinib treatment. It is known that there are multiple pathways in which expression of the BCR::ABL1 tyrosine kinase causes genetic instability with subsequently emerging mutations. The resulting adverse response to treatment may no longer depend on the BCR::ABL1 kinase itself [40].

In a minority of patients (5/100; 5%) mutations in addition to *BCR::ABL1* persisted over all time points analyzed and therefore seem to precede the *BCR::ABL1* translocation indicating a multistep pathogenesis in CML as suggested previously [6, 41, 42]. One mutation affecting *CALR* displayed slightly increased VAFs in follow-up samples despite successful suppression of the *BCR::ABL1* clone. *CALR* mutations are known drivers of myeloproliferative neoplasms [43] and may therefore contribute to a clonal advantage and expansion even after the *BCR::ABL1* clone has been eradicated. Four *DNMT3A* mutations showed stable lower VAFs in follow up samples compared to the respective diagnosis sample. A suppression of the mutation, but not eradication despite good response, indicates the existence of a preleukemic clone that subsequently acquired *BCR::ABL1*. *DNMT3A* is known to be frequently mutated with increasing age in the general population as part of a phenomenon termed clonal hematopoiesis of indeterminate potential (CHIP) [10–12]. In elderly people without hematologic disorders *DNMT3A* mutant clones are unlikely to expand over time [44], which supports the finding of stable VAFs under TKI therapy in CML patients. Age-related clonal hematopoiesis is associated with an increased risk of developing hematologic cancer [11]. In the study presented here, median age of patients carrying additional mutations at diagnosis was 54 years (range, 22–78 years) whilst mutation frequency was 24%, thus exceeding the previously established age-related mutation prevalence of <5% within the healthy population in this age range [11]. Whilst most detected mutations are likely a direct feature of CML pathology, the *DNMT3A* mutations in particular may have occurred as age-related events which might predispose individuals to CML. The precise implication of preleukemic clonal hematopoiesis for CML has been analyzed in a few studies but will undoubtedly be better understood as sequencing efforts expand [6–8]. Similarly, in 14 patients germline mutations were detected which met the published criteria of clinically relevant mutations [8]. Five of the affected patients also carried at least one somatic mutation. This indicates that germline mutations may also function as predisposing factors to CML.

## Conclusion

The study presented here aimed to evaluate the prevalence, dynamics and clinical impact of *BCR::ABL1* independent gene mutations in chronic phase CML patients treated with nilotinib in an ongoing prospective clinical trial. Mutations in addition to *BCR::ABL1* were frequently identified at diagnosis and were found to vary in their dynamics during treatment. *ASXL1* mutations were most common and affected patients showed MMR less frequently at month 12, 18, and 24 compared to all other patients. *ASXL1* may, after further validation, serve as an additional prognostic factor for molecular response in newly diagnosed CML patients.

## Supplementary information

Supplementary Table 1
